# Supporting Family Caregivers in an Aging Society: The Need for Comprehensive, Evidence-Based Interventions

**DOI:** 10.3390/ijerph22121826

**Published:** 2025-12-05

**Authors:** Hanne Konradsen, Zarina Nahar Kabir

**Affiliations:** 1 Department of Geriatrics and Palliative Care, Copenhagen University Hospital Amager and Hvidovre, 2650 Hvidovre, Denmark; 2 Steno Diabetes Hospital Copenhagen, 2730 Herlev, Denmark; 3 Department of Neurobiology, Care Sciences and Society, Karolinska Institute, 171 77 Stockholm, Sweden; zarina.kabir@ki.se

Globally, the population is aging, representing one of the most significant societal transformations of our time. By 2025, the population aged 65 years and over is expected to have increased from the current 703 million to 1.5 billion [1], a change that brings with it unprecedented challenges in providing adequate care and support for older adults. In parallel, there is a similar increase in the prevalence of chronic disease, with many conditions becoming more prevalent with age [2]. Therefore, the simultaneous increase in pressure on families, healthcare systems, and societies at large may lead to major challenges in a relatively short timeframe.

Most care for older adults is provided by family caregivers. In low- and middle-income countries, this is often due to social norms or limited publicly funded formal care, while in high-income countries, it is often complementary to formal care services [3]. These informal caregivers—spouses, adult children, siblings, and other relatives—form the basis of long-term care across the globe. Assisting with activities of daily living, medication management, emotional support, coordination of medical appointments, and much more, they are often the ones who enable older adults to age in place.

For many family caregivers, caregiving brings genuine satisfaction. The opportunity to reciprocate care received earlier in life, to spend meaningful time with loved ones, and to honor cultural and familial values of intergenerational support can be deeply fulfilling. Research has documented the positive aspects of caregiving, including enhanced feelings of purpose, strengthened family bonds, and personal growth [4,5]. These experiences should not be diminished or overlooked in our discussions of family caregiving.

However, we must also acknowledge the risks and challenges informal caregivers face, particularly female caregivers and especially when care needs become complex and prolonged [6,7]. The demands of caregiving often collide with other life responsibilities in ways that create significant stress and burden. Many caregivers struggle to maintain employment while providing care, leading to reduced work hours, career disruptions, or complete workforce withdrawal [8]. The challenge of balancing caregiving and personal participation in social activities and work responsibilities can lead to isolation, financial challenges, and reduced quality of life [9]. Furthermore, family caregivers often neglect their own health, potentially reducing their care capacity and impacting their well-being.

These converging challenges create a situation in many families worldwide, where they risk increasing caregiving burden with insufficient support. It is therefore of growing importance to better understand how we can support family caregivers and develop interventions that enhance their caregiving capacity while protecting their well-being.

Addressing the needs of family caregivers requires moving beyond an acknowledgement of their contributions to the development and implementation of effective, evidence-based interventions. This Special Issue responds to this imperative by bringing together research that advances our understanding of family caregiving dynamics and evaluates interventions designed to support caregivers.

A comprehensive understanding of families’ daily living and what types of support would make the most meaningful difference in their lives is also essential. This calls for a dual approach to understanding the situation for caregivers. As older people are increasingly living with multimorbidity, greater information on support in general is key, as well as an awareness of the specific challenges of, for example, caring for a parent with dementia versus caring for a spouse with heart failure or a sibling with mobility limitations. As the trend of increased migration for a number of reasons, e.g., career opportunities, conflicts, economic vulnerability, climate change, and so on, can be seen worldwide, we must consider that family caregivers will likely also migrate, and we must therefore understand how caregiving experiences vary across cultures, socioeconomic groups, geographic settings, and family structures.

Research must also examine the temporal dimension of caregiving. The needs of someone newly entering the caregiver role differ from those of someone providing care for many years. In addition to this, care transitions—such as hospital discharges, changes in care settings, or shifts in the older adult’s functional status—represent particularly vulnerable periods that warrant specific attention and support [10].

## 1. A Multi-Level Approach to Intervention

The delivery of effective support for family caregivers requires interventions at multiple levels: systemic, relational, and practical. Each level addresses different aspects of the caregiving experience, together forming a comprehensive approach to caregiver support.

At the *systemic* level, there is a requirement for policies and programs that recognize family caregivers as essential partners in health and social care, including the development of caregiver assessment tools that identify caregiver needs and risks as part of routine care planning. Healthcare systems should also implement systems for including family caregivers in care pathways, and a particular focus on immigrant family caregivers is essential to ensure an inclusive health and social care system [11].

Workplace policies play a crucial role in enabling employed family caregivers to balance their responsibilities. Paid leave, flexible work arrangements, and caregiver-friendly workplace cultures can make the difference between sustainable caregiving and crisis. Some countries have implemented innovative programs such as caregiver allowances [12] or designated social support to family caregivers within the existing health and social care system [13,14].

The education of healthcare professionals must also continually develop their training on working with family caregivers, recognizing their expertise, assessing their needs, and supporting them [15].

At the *relational* level, interventions focused on the interpersonal dynamics of caregiving and the quality of relationships within the family system are essential. Family support can help families navigate difficult care decisions, resolve conflicts, and distribute caregiving responsibilities more equitably [16].

Training in care management can help caregivers navigate the emotional challenges inherent in family caregiving, with some innovative programs focusing on enhancing the quality of the dyadic caregiver-care recipient relationship through shared activities, reminiscence therapy, or mindfulness-based interventions that strengthen connection and meaning-making.

At the *practical* level, caregivers require concrete skills, information, and resources to provide effective care while protecting their own well-being [17]. These programs must be accessible, culturally appropriate, and tailored to specific conditions and care situations.

Technology-based interventions, including telehealth consultations, remote monitoring systems, and caregiver support apps, can enhance the ability of caregivers to manage care from a distance and connect with professional support when needed [18,19].

Developing and testing effective interventions is not enough; ensuring their implementation and sustainability presents an equally important challenge. Research must examine barriers and facilitators to implementation, identify strategies for adapting interventions to local contexts while maintaining fidelity to core components, and evaluate implementation outcomes alongside effectiveness.

Sustainability requires attention to be given to economic evaluations and integration with existing services. Interventions that remain dependent on a temporary grant are not sustainable.

## 2. A Call for Multidisciplinary and Multinational Research and Collaboration

Addressing the complex needs of family caregivers requires multidisciplinary collaboration. This Special Issue seeks to present a collection of unique perspectives that enrich our understanding.

Looking forward, we particularly encourage research that focuses on the following elements:

- The examination of caregiving experiences across diverse populations and cultural contexts;
- The evaluation of the effectiveness and cost-effectiveness of caregiver interventions;
- Exploration of the intersection of caregiving with employment, education, and other social roles;
- Investigation of the long-term health impacts of caregiving and protective factors;
- Analysis of policy approaches to supporting family caregivers across different healthcare systems;
- The development and testing of technology-based solutions for caregiver support;
- The study of care transitions and how to improve continuity and coordination;
- The examination of ethical issues in family caregiving, including decision-making, autonomy, and caregiver burden.

The aging of our global population is inevitable; the burden experienced by family caregivers is not. Rather than viewing formal and informal care as separate systems, integrated models are required where family caregivers and professional care providers work as partners, each bringing their unique knowledge and capabilities. A shift from a crisis-response approach to preventive support is called for that identifies caregiver needs early and intervenes before the burden becomes evident.
